# Collectanea

**Published:** 1829-11

**Authors:** 


### COLLECTANEA.

Floriferis ut apes in saltibus omnia libant,
Omnia nos, itidem, depascimur aurea dicta.

### PHYSIOLOGY.

*Experiments which clearly prove that the Nervous Tissue possesses the property of developing the Galvanic Fluid.* By Dr. Louis Beraudi, of Turin. (*Annali Universali di Medicina*, 1829.)

The numerous experiments of Wilson Philip, Edwards, Vavasseur, Aldini, Magendie, Krimer, and Wienhold, establish the fact that the nervous system developes galvanic phenomena. Dr. Beraudi has instituted a series of experiments for the purpose of collecting the galvanic fluid that is thus formed, that no doubt may any longer attach to so curious a fact. The following are the results of these experiments.

1st. Dr. B. exposed the right crural nerve of a living rabbit, the temperature of the apartment being raised to 15° Réaumur. After having carefully removed all the blood, three small and very fine steel needles were introduced into the nerve: they were separated by a small stick of sealingwax placed horizontally. The animal evinced great pain, and at the end of a quarter of an hour it was found that the needles had not acquired the power of attracting small pieces of paper. The same needles were again introduced into the nerve, and, upon being withdrawn at the expiration of a quarter of an hour, as before, each needle was found slightly to attract small particles of rust of iron, whilst the bits of paper remained unmoved.

2d. The same experiment was repeated, on the same day, on another rabbit, but without a similar result. It had been observed that the developement of the electric fluid diminished in a direct ratio to the slowness of the circulation. Pulmonary insufflation was therefore had recourse to, and at the expiration of ten minutes the magnetic property of the needles was very manifest. From this fact Dr. B. concluded that the strength of this property of the needles, produced by the nervous tissue, was in proportion to the greater or less quantity of blood exposed to the contact of the air. This remark was communicated to Professor Rolando, who recommended Dr. B. to vary the experiment, by causing the animal to respire different gases.

3d. The apartment being of the same temperature as above mentioned, it was found that, by introducing into the lungs oxygen, hydrogen, and azote, the magnetic property developed in the nerve was very powerful after the insufflation of the first of these gases, weaker from that of the second, and not at all apparent from that of the azote.

4th. After having divided the spinal marrow of a rabbit, between the third and fourth cervical vertebræ, needles were introduced as before into the crural nerve; but in neither was the magnetic property detected, until a certain quantity of oxygen had been introduced into the lungs: it then was very manifest.

5th. The right optic nerve of a rabbit was exposed, and one needle introduced into it; which was withdrawn in eight minutes, and exhibited no magnetic property. The animal was then made to respire oxygen gas, by means of a bladder which was filled with it; but still no magnetic effect was produced. Neither hydrogen nor azotic gas produced any effect. At the expiration of an hour, the same needle was introduced into the crural nerve of the same rabbit. The animal was again made to respire oxygen gas, and a weak magnetic property was then perceptible in the needle; which was no longer developed when the experiment was repeated after having divided the spinal marrow at the part above mentioned.

6th. The same experiment was repeated, in presence of Professor Rolando, upon the olfactory nerves, but without any result.

7th. A ligature was placed upon the crural nerve of a rabbit, into which needles were introduced *below* the ligature. No galvanic phenomena were produced. The same was also the case after the division of the nerve.

8th. Dr. B., after the manner of M. Vavasseur, endeavoured to ascertain whether this property, which was imparted by the nerve, could be communicated at any distance. To determine this fact, the crural nerve of a rabbit was laid bare and divided; and the extremities of the nerve then separated to a distance of about four lines. A needle was placed in the inferior portion of the nerve, and it was found that it had, in a minor degree indeed, acquired the magnetic property. This result confirmed Dr. B. in the opinion that the nervous influence is developed at some distance, which he had before presumed from finding the magnetic property of the needle diminish and disappear from the inspiration of hydrogen and azote.

As all philosophers believe that the galvanic fluid is capable of imparting to iron a magnetic property, and as the identity of these two fluids is admitted, the following results are deducible from the above experiments:

1. Electricity developes itself in the nervous system.
2. The fifth and sixth experiments confirm the theory of Prof. Rolando.
3. Respiration appears to have considerable influence upon the developement of the galvanic fluid in the nervous system.
4. It may be presumed that the galvanic fluid does not emanate from every part of the nervous system, but perhaps from the cerebellum, as Mr. Rolando supposes.

Lastly. That neither the olfactory nor optic nerves concur in the developement of that fluid.

Dr. Beraudi does not claim the merit of first conceiving these experiments. He is aware that Beclard had before ascertained and announced, that a needle becomes magnetic from being introduced into a nerve.

---

*Malformation of the Œsophagus and Stomach in an Infant.* By Dr. Pagenstecher. (*Archives Générales.*)

A woman, named Godvin, having passed through the proper period of pregnancy without any accident, was delivered of a full-grown and externally well-formed female child. It was soon found that the infant could not swallow, and that after a few seconds it threw up the milk it had taken. For four days the life of the child was supported by milk clysters, but at the end of that time it died. Upon opening the body, the œsophagus was seen to terminate in a cul-de-sac, near the bifurcation of the trachea, about fourteen lines below the pharynx. A small cellulo-fibrous band arose from the anterior part of the cul-de-sac, and terminated in the extremity of that portion of the œsophagus which was attached to the stomach, and which was about one inch and nine lines in length. The structure of the stomach was porous. Its mucous and muscular membranes formed a kind of net, the meshes of which were closed by the peritoneal tunic, which was itself pierced in different places. The meshes were from a quarter of a line to a line in diameter, and were more than a hundred in number.

This case refutes the opinion of those physiologists who explain the nutrition of the fœtus by the deglutition of the water of the amnion, which was in this case obviously impossible.*

---

*Account of a living Duplex Child.* Communicated by CHARLES BELL, F.R.S. to whom the letter is addressed. (*Medical Gazette.*)

My dear sir, *Teddington; 17th Sept.* 1829.

A recent excursion to Switzerland gave me occasion to see, on the 1st of August last, at Geneva, a remarkable example of a living *lusus naturæ*, or monstrosity in the human frame: namely, twin infants furnished with two heads, two necks, and four arms, but grafted or united side to side, so as to form only one female body, terminating in two legs, or inferior extremities of usual shape. This phenomenon presents nothing disgusting to the beholder: on the contrary, the intelligence which already begins to develope itself in the heads, makes it an object of great interest. I had not the opportunity of a very minute personal examination, in consequence of only seeing it at the hour of its daily exhibition to the public; but my observation verified the accuracy of the subjoined description, by Mons. F. MAYOR, which was published in the *Journal de Genève* of the 30th of July:

Marie-Terèse Parodi, thirty-two years of age, the mother of several perfect children, gave birth, on the 12th of March of the present year, to a double child, now 140 days old. The one to the left was baptized by the name of Christina, the other by that of Harriet.

At the first glance it is perceived that twin infants have become grafted together: however, when they are regarded from before, the lower parts of the body appear simple from the stomach downwards, while the chest is divided at its upper part, at least on one side of the trunk. A more attentive examination speedily enables us to recognise the following peculiarities: Anteriorly, the chest only appears to form one thorax; the sternum forms a kind of gutter at its inferior part, while above it widens and enlarges very much, in order to give attachment to four well-formed clavicles, two of which are fixed at the external angles of that bone, and the other two at the middle of its superior border. Each of these four clavicles is directed towards one of the shoulders, and gives all the support necessary for the movements of the arms, of which two are placed between the heads. The right edge of the sternum appears to give attachment to the right ribs of Harriet, and the left to the corresponding ribs of Christina. There are four mammæ; the two in the middle being smaller than those which are external to them, and are encroached upon by the armpits of the middle set of upper extremities. There is but one umbilicus. When the examination is made from behind, two spinal columns are distinctly seen, sufficiently separated from each other at the upper part of the body; but they approximate towards the sacrums, of which there are two, united by the left edge of the one and right of the other, in such manner, however, that the ossa coccygis are quite distinct. From each

* How was the fœtus nourished, which was born alive, and which had neither funis nor umbilicus? From this remarkable case, MASON GOOD was led to infer that the "liquor amnii is the proper source of nourishment to the fœtus." Vide London Med. and Phys. Journal, p. 167, Feb. 1829.—EDITORS.

vertebral column there arise ribs, which are directed towards each other: the four or five first run to the anterior sternum; but the rest are united to those of the neighbouring body, at least by their external surface, and appear only to form one circle with those of the anterior part of the trunk. Thus, then, the thoraces are really separated externally throughout their upper third and probably entirely so within: the posterior ribs of this double trunk participate in the movements of respiration, in the same way as those of the anterior part. The beating of the heart in Christina is perceived at the anterior and left surface of the trunk; the beating of the heart in Harriet is seen at the middle part of the posterior surface. Beneath the ribs of this same side there is, between the two spinal columns, an abdomen twice as small as that on the anterior surface of the trunk. Harriet has had from her birth some malformation of the breast, for it is not long since the blueness with which she was affected began to disappear. For some days she has had a catarrhal affection, and her pulse was at 168 in a minute, while her sister enjoyed perfect health, the pulse not exceeding 144 in the minute. Their breathing is not always synchronous: however, there is reason to believe that a communication exists between the lobes of the lungs of the two children. The one sometimes sleeps while the other is awake; sometimes sucks while the other plays, or wishes also to get the breast; but never has one an evacuation without the other making the same efforts, which even wake her, if asleep. As they grow older, other and yet more interesting phenomena will doubtless be observed.

Examples of this kind of union are happily but little common, and it is but rarely that they survive their birth. A good many cases, indeed, are mentioned by authors, but most of them are apocryphal; some, however, are well authenticated: such, for example, as the two Hungarian girls, spoken of by Buffon, who were united by the loins, and who lived twenty-one years. Another case of a similar kind occurred at Verdun, in 1709: here also two females were united, and in the same manner; they were then seven years of age, and could walk; and their intelligence was so great that they had acquired several languages. There is also an instance in which two little girls were united from the lower part of the sternum to the umbilicus. The accoucheur divided the parts, and, thus separated by an operation, the children lived.

In 1495, there were born, near Worms, twins united by the forehead: they lived for ten years, when one died, and the other was separated by an operation; but it proved unavailing. In 1525 a native of Savoy, thirty years of age, and of the ordinary stature, exhibited himself. He had hanging from the sternum a body about a foot in length, having feet and arms, but without motion, while the head appeared as it were planted in the body of the man. In 1538 there was in Bavaria a female mendicant with two heads, who was driven from the country lest the pregnant women should give birth to similar monsters; a fear as imaginary as the result of it was cruel and uncharitable. Buchanan, in his History of Scotland, mentions the case of a monster with two heads, which lived twenty-eight years. The two heads, having different volitions, often quarrelled. They both felt wounds of the lower part of the body, but those of the upper part were only perceptible to the corresponding head. In 1552 a French woman, at Geneva, was brought to bed of a monster, the heads of which were united by the posterior part, and the union extended to the lower part of the back. Gaspard Materier took a portrait of it. The monster lived some hours, and is compared to Janus by a writer (Lycosthene) who describes it.

Before we conclude, we may allude to the opinion, which has been frequently started and recently renewed, that such monsters ought to be destroyed immediately after their birth. *No one can have a right to do so;* for, since God ordains that such beings should come into the world, the laws owe them protection. Besides, it would be very difficult to determine the degree of imperfection at which an infant would cease to have a right to live; for these phenomena are met with from a simple supernumerary tip of the ear up to the example above mentioned, of two girls who were successfully separated by an operation.

The catarrhal affection, with febrile excitement, under which the twin named Harriet laboured on Thursday the 28th July, noted by Mr. Mayor, had subsided on the 1st August, and she then had an appearance as healthy and lively as Christina. Both infants seemed to exercise some control over the motion of the lower limbs; but, should they live until their mental faculties and animal powers are further developed, it will become a matter of curious inquiry to ascertain in what manner nervous influence, springing from two distinct organs of sensation and volition, shall be directed towards the lower extremities, so as to effect locomotion in accordance with the will of each sensorium; or whether there shall be occasional contentions between the *heads* for a dominating power over the *legs.*

Although the precise peculiarities of structure in the abdominal viscera, and the question as to whether the internal organs of generation correspond in unity and simplicity with the external, are points which cannot be fully determined till after death; yet, from the circumstance of each infant taking food with avidity at different times, it may be inferred that each has its proper stomach, and that the union of the alimentary canal takes place below that organ.

Many facts desirable to be ascertained hereafter, during the growth of this extraordinary animal phenomenon, must arise, referring especially to anatomy and physiology; and, as you have been long an eminent professor of these branches of medical science, I am induced to address to you this letter, in the belief that it may invite you to gratify your own zeal, and to indulge that of the profession, by instituting further inquiries, from time to time, regarding the interesting subject of it.

I remain, my dear sir, very faithfully yours,

J. Borland.

---

### PATHOLOGY.

*Metastasis of Leucorrhœa.* By Dr. Wassenfuhr. (*Rust's Mag.* t. xxvii. 2e cah. 1828, p. 295.)

A young lady, mother of several children, had been affected with leucorrhœa for several years, which resisted every treatment that had been employed. Dr. W. prescribed baths and aperients, and at the same time injections into the vagina of corrosive sublimate. On the third day the discharge from the vagina had entirely ceased, but a precisely similar discharge issued from the mouth. It resembled that which had appeared from the vagina, in consistence, colour, and quantity. The mucous membrane of the mouth was redder than natural, swollen, and very painful. It resembled the appearance which is seen in cases of aphthæ. The diagnosis, however, was not difficult; and the lady requested the disease might be removed to its original seat! In eight days this object was effected, and the discharge of the mouth speedily subsided.

---

*Pustules and Vesicles on the Tongue in Hydrophobia.*—Dr. F. Fuchs, of Rapperschwyl, relates a case of hydrophobia, (*Rust's Magazin*, tome xxvii. 3e cah. 1829, p. 514,) in which the appearances upon the tongue corroborate the statements of previous observers. Upon the *upper* surface of the tongue, near its base, were forty or fifty small brown pustules, regularly disposed upon each side of the organ. These pustules were rather prominent, and covered by a thick and tough envelope. Most of them had a black point in the centre. They were very distinct from the natural papillæ of the tongue. In other respects the tongue appeared healthy. Neither the submaxillary nor other glands were swollen.

In another case, a crowd of vesicles were seen upon the base of the tongue, the largest of which were about the size of a pea. They contained a thick lymph.

---

*Vaccination from the Cow.*—Some cows, in Hyde park, were recently affected with an eruptive disease on the udder, supposed to be cow-pock. Two attempts were made to propagate the disease to the human subject, but in both instances the experiment proved inefficient. In one case, no apparent effect was produced; and in the other only a transient and incomplete papulation resulted, and which died away without running on to vesication. At the time we saw the disease in the cow, it had passed into the form of crusts, from beneath which oozed a certain quantity of purulent matter. The genuine vaccinia in the cow would appear to be a rare disease in this part of England: several attempts, similar to the above, have been made here, with an equally unsatisfactory result.—*Med. Gazette.*

---

### SURGERY.

*Extirpation of Diseased Ovaria.* By Dr. Hopfer. (*Graëfe und Walther's Journal fur Chirurgie*, tome xii. 1er cah. 1828, p. 60.)

Three cases are related. One woman survived and bore a living child after the operation. The diseased ovarium which was extirpated weighed eight pounds. The two other patients died a few hours after the operation. Dr. Chrysman appears to have been the first surgeon who performed this operation in Germany.

---

*Lithotomy, à deux temps.* (Gibson's *Medical Sketch of Dumfriesshire.*)

I was present when Mr. Lizars, of Edinburgh, performed the operation of lithotomy in this town, during the present summer. It was speedily and simply done. One calculus, the size of a pigeon's egg, was easily removed, as soon as an opening had been made into the bladder; when another was discovered, somewhat larger than the first, but, owing to the firm contraction of the fibres of the wounded bladder, it could not be readily removed at the time, and Mr. Lizars put his patient to bed; assuring his medical friends that all further attempts to remove the calculus would only tend to bruise and irritate the bladder and adjacent parts, and render inflammation more liable to occur.

He was confident, he stated, from experience, that, on the third day from the operation, the calculus would be easily removed, with scarcely any pain to the patient. Accordingly, on the day appointed, those who were present at the operation were in attendance, and saw Mr. Lizars gently introduce his finger into the wound, while the patient lay in bed, and then guiding a scoop along his finger, bring out the calculus, which was as large as a chicken's egg, with all the ease imaginable. The patient, a gentleman of sixty-four years of age, had a quick recovery.

Mr. Lizars speaks highly of leaving the calculus till the third day, when it cannot be readily extracted at the time of the operation. By that time the suppurative process has commenced, and all the parts concerned are quite relaxed. This is the method introduced by the French surgeon FRANCO, as the "*operation à deux temps*," and which has been condemned by some of our modern writers.

Mr. SAMUEL COOPER strongly reprobates the practice of putting a patient to bed with a stone in his bladder; and advises that, rather than do this, we should make an opening adequate for its extraction; or, if this cannot be done, he tells us to break down the calculus, and remove its fragments. If the long and constant irritation of a calculus, or calculi, has the effect of thickening the coats of the bladder, and diminishing its capacity, and if the cutting into that viscus causes its fibres to contract and firmly grasp the calculus, as the uterus does its placenta when about to throw it off, (both of which occurrences experience shows us to be almost invariable attendants on this disease, and the operation for its removal,) then all reiterated and painful attempts to remove or break down the calculus will not only be improper, but must also tend greatly to endanger the life of the patient.

The cases in which Mr. Lizars has tried this operation *à deux temps* have been attended with the greatest success, and he has removed, on the third day after the operation, very large calculi, with the utmost ease. He has hitherto made one or two gentle endeavours to bring away the calculus at the time of the operation, but, if he does not readily succeed, the patient is put to bed. So convinced is this expert operator of the superiority of this plan, that he has declared to his medical brethren, at the operation I have just mentioned, that, were it his misfortune to be obliged to submit to the operation of lithotomy, he would not suffer the forceps or scoop to be used before the third day.

---

*Lithotrity.*—M. SEGALAS recently communicated a case to the Académie Royale de Médicine, in which lithotrity was practised successfully upon a little girl, only three years old.—*Archives Générales.*

---

*Académie Royale de Médecine. New Mode of arresting Hemorrhage.*

M. AMUSAT recently described to the Academy his new mode of arresting hemorrhage from large blood-vessels, without ligature, compression, or any of the means previously resorted to. He ascertained, from a series of experiments, that the laceration or contusion of large vessels in most cases produces but a momentary suspension of hemorrhage, and that it is *permanently* arrested by a methodical contortion of the bleeding vein or artery. He suggests the following plan.

The vessel, being seized with a pair of forceps, the branches of which are firmly fixed by a screw, is extracted so as to be denuded for about six lines. After having been freed as much as possible from the surrounding cellular tissue, it is held between the forefinger and thumb of the left hand, whilst the forceps are twisted five or six times, according to the size of the vessel, until the portion between the teeth of the instrument is lacerated. The artery then spontaneously retracts, and is seen and felt to pulsate, though the bleeding is completely stopped. Upon examining the vessel, it appears that the internal coat is divided as by a ligature; that it contracts, and forms a kind of circular pad, by which further hemorrhage is obviated. It is important that the extremity of the vessel should be fixed between the fingers of the left hand, or else the contortion will extend through the cellular tissue as far as the next collateral branch.

Mr. A. informs us that, in many experiments on rabbits, dogs, and horses, the most complete success has resulted from this plan. In one case of contraction, and another of amputation of the thigh, in the human subject, it has also succeeded. He likewise tried its effect upon ossified arteries in the dead body, and found it succeed; but he doubts whether this would be the case during life. In horses, although the parietes of the vessels are of considerable strength, M. Amusat arrested hemorrhage both from the carotid and jugular vein. To determine the value of this novel plan in comparison with others, he several times tied the crural artery on one side in dogs, and contorted that of the other side. In two cases where fatal hemorrhage ensued, it took place from the side where the ligature had been employed. In the first place, this mode of proceeding has the merit of great security. It is said also to possess many advantages over other methods, from its greater facility. It admits of immediate reunion of the wound, and is readily applicable in many cases where it would be difficult to apply ligatures without the risk of including other organs.

---

### M. Levert *on Metallic Ligatures applied to Arteries.*

Some years ago Dr. Physick suggested the propriety of an animal ligature, thinking that it would be removed by the absorbents: the external wound might therefore be closed, and all the bad effects produced by the ordinary ligatures thus obviated. We cannot say positively what has been the result of this practice, but believe that the animal ligature is not used so much as its importance demands.

The same gentleman has likewise suggested the use of leaden ligatures, with the view of obtaining such results as were hoped for from his animal ligature, or the temporary one of Dr. Jones. To this he was led by a knowledge of the fact that bullets, buckshot, and lead, would remain in contact with almost any tissue of the body, without producing irritation or unpleasant consequences, and that for an indefinite period. So far as I know, a trial of this ligature has never been made: with a view, therefore, to ascertain its effects, I have instituted a number of experiments, the results of which I will now relate.

Experiment I.—On the 16th of March, 1828, I laid bare the right carotid artery of a dog, and, after separating it carefully from its accompanying nerve and vein, I passed under it a lead wire, and tied it firmly. Both ends of the wire were then cut off with a pair of scissors, and the sharp points bent down with a common dissecting forceps. The wound was now drawn together with a few stitches of the interrupted suture, and over these were laid some adhesive strips. This animal was not confined, but suffered to run at large. When I examined him several days after, I found the stitches ulcerated out, and the wound open; it had filled up from the bottom with granulations, but the edges of the skin were separated to a considerable distance. With light dressings, it healed entirely by the 5th of June.

June 28th.—I killed this animal, and dissected with care the neck. A small cicatrix existed in the skin; the lead was found in the situation in which I had placed it, by the side of the vein and nerve, perfectly encysted. The artery at this place had been removed entirely, for the space of half an inch.

Both ends of the vessel, caused by this removal of its central portion, adhered by loose cellular substance to the surrounding parts, which appeared to be in a perfectly natural state. The end towards the heart was not at all increased or diminished in size; it was sealed up for three eighths of an inch in extent, by an organised substance, resembling a coagulum of blood in colour, but not in consistence, it being much firmer. The end towards the head resembled the one just described, in all particulars: the substance, however, which filled its extremity was of greater extent, and occupied the whole space up to the next branch, which was rather more than half an inch.

Not the slightest trace of inflammation existed in the neighbouring parts: on the contrary, they appeared perfectly natural. The lead itself was enclosed in a dense cellular substance, which formed for it a complete cyst.

Experiment II.—The right carotid artery of another dog was separated from its contiguous parts on the 17th of May, and a lead wire placed around it, as in Experiment I. The lips of the wound were kept in contact with sutures and adhesive strips. I examined it three days after, and found that it had united by the first intention in the whole of its course, except in those points included by the stitches: these I cut loose, and dressed it simply with adhesive strips. When I looked at this dog again, I found that, from the itching of the wound, the animal had scratched off the dressings, and broken up the new adhesions: I washed it carefully to remove the dirt, and dressed it with simple dressings. It healed kindly, and was entirely well on the 6th of June, at which time I killed the dog, and made a careful dissection of the parts. The cellular substance here was much thickened and indurated, forming a strong bond of union between the nerve, vein, and artery. The two former were in their natural condition; the artery was pervious its whole extent, to within three eighths of an inch of the wire. At this place the caliber was entirely obliterated; a firm substance, resembling bruised muscle, filled its cavity. Between the ligature and the head the artery was impervious, and much diminished in size, having the appearance of a mere cord, not exceeding one fourth the original dimensions of the vessel. The lead preserved its situation around the artery; it had become entirely encysted, and not the slightest remains of inflammation existed.

Experiment III.—I cut down on the left carotid of a third dog, on the 29th of May, and proceeded as in Experiments I. and II., differing in no respect, except in dressing the wound: I used no stitches, but merely adhesive plasters.

June 1st.—I examined the wound, and found that it had united through its whole extent; but, as I supposed the union not to be very firm, the strips were reapplied, and suffered to remain on until the 5th, when they were removed altogether.

June 27th.—The animal was killed, and a minute examination made. The lead wire was found around the vessel, which was impervious for an inch or more, as in the former experiments. The surrounding parts healthy.

Experiment IV. June 9th.—The dog which was the subject of the last experiment, having entirely recovered from the first operation, now became the subject of a second, which was performed on the carotid of the opposite side. This was conducted exactly as the preceding, the wound united by the first intention without the least difficulty; no constitutional symptoms manifested themselves.

On the 27th, at which time this dog was killed, an examination was likewise made of this side of the neck: the appearances corresponded exactly with those of the preceding experiments.

Experiment V. August 5th.—I performed a similar experiment on the carotid of another dog. I killed him on the 3d of September, and found that the appearances differed in no respect from the foregoing.

The lead having answered my expectations so well in these cases, I felt a great inclination to ascertain whether that substance alone possessed the property of remaining in contact with the living tissues, without exciting irritation or any unpleasant consequences, or whether similar results might not be obtained by using the other metals. I accordingly continued the subject, using gold, silver, and platinum, instead of lead.*

From the experiments now detailed, we may, I think, conclude that the plan of tying arteries with lead and the other metals is free from danger, and may be productive of some peculiar advantages: more experience, and a greater number of experiments, are necessary to establish this point thoroughly, and it is to be hoped that some one, fully competent to the task, will prosecute the subject.—*American Journal of Med. Sciences.*

---

*Improved Method of treating Laceration of the Perineum.* By Dr. Dieffenbach, of Berlin. (*Chirurgische Erfahrungen, besonders über die Wiederherstellung Zerstörter Theile, &c.* Berlin.)

Dr. D. has turned his attention chiefly to the worst description of cases, where the rent extends along the whole perineum, so as to make a communication between the rectum and vagina, and render the patient incapable of retaining the feces. It has been generally conceived that the operation for reunion of the lacerated perineum is undertaken with the fairest prospect of success during childbed, because the parts have not acquired that callosity and indolent character which they assume when of old standing. Dr. D., however, opposes this practice, and maintains that it has appeared more successful than the contrary system, only because the lacerations which are commonly healed in this way are of insignificant extent, and will on that account heal at any time. In the instance of an extensive laceration which connects the rectum with the vagina, he urges that, if we take into consideration the exhaustion the patient must have endured from the kind of labour by which alone so severe an injury could have been produced; the impossibility

* We do not give the various experiments made by Dr. L. with these metals, as the results were materially the same as from the use of lead.—Ed.

Quite enough torture & killing of making her preserve, in her debilitated state, for an adequate length of time, the position requisite for the cure; the obstruction which the healing of the wound will experience from the flow of the lochia, and the preternatural secretion of the vaginal mucus for some time after delivery; and the contused nature of the wound which is to be united; it must be preferable to wait till the patient's health is restored, even although the sides of the wound should become indurated, because there is no difficulty in giving them, with the knife, surfaces much better fitted for adhering than the original surfaces of the laceration.

He proposes that, before the operation is begun, the intestines shall be thoroughly cleared out by laxatives and clysters; after which opium is to be given, and repeated at intervals, to prevent any discharge from the rectum for eight days at least.* The surfaces being brought into the state of a fresh wound by the removal of their indurated exterior, they are to be brought closely in contact at the centre by a strong knotted suture, which is introduced in such a manner as to pass through the loose cellular tissue at the bottom of the wound; two small needles, with twisted sutures, are then to be introduced through the lips of the wound, on the vaginal side of the principal suture; the little slit in the rectum itself is then to be united by two knotted sutures, introduced with small stitching needles; and two twisted sutures are lastly passed through the lips of the wound, between the rectum and great central sutue. The ligatures and ends of the needles are cut away as close as possible.

The most important part of the operation still remains. This is the dividing of the integuments on each side of the wound, by an incision running at the distance of half an inch, in nearly a parallel direction with it, but curved a little outwards at the middle. These incisions are no sooner made than the stretched state of the newly united parts at once ceases; the central wound retires backwards, so that the lateral cuts appear like free incisions or steps with sharp edges, and the slight movements of the patient, which it is impossible to prevent altogether, have no influence on the sutures or the adaptation of the surfaces.

The treatment consists in the constant employment of cold poultices for the first few days, the careful cleaning of the parts by squirting warm water, restriction to a low diet, *and the maintenance of the constipated state of the bowels for eight days.* A T bandage, or any more complicated bandage; a pessary, or sponge, in the vagina, or any of the other mechanical means resorted to for maintaining the parts in a state of rest, is unnecessary by this mode of operating, and many of them cause inconvenient irritation. The removal of the urine by the catheter may be advisable, but is unnecessary if the hair has been shaved away from the parts before the operation. About the fourth or fifth day, lead lotions may be substituted for the cold poultices, and charpie placed on any spots which are suppurating. The sutures may be gradually withdrawn between the fifth and tenth days.

Not unfrequently, although the parts have united generally, a small rent is left in the rectum. This, however, is commonly found to heal up gradually,

* This precaution is important. A case recently occurred under our own observation, in which the newly and but partially united parts were torn asunder during the operation of a small dose of castor oil, which was imprudently given too soon.—EDITORS.

by the careful use of stimulant applications to encourage granulation. The lateral incisions require no particular attention, as they are covered by the poultices in the early period of the treatment, and may be dressed with charpie afterwards till they heal up; which commonly happens in fourteen days.

Dr. D. describes minutely a case in which this plan was completely successful. In eight days every part adhered firmly, except a small fissure in the rectum, which was also filled up by granulation before the end of the fourth week. The patient, a servant, who had been reduced to a state of great misery and helplessness in consequence of the unceasing involuntary discharge of flatus and feces, was thus restored within a month to a state of perfect health.—*Edinburgh Med. Journal.*

---

*Extirpation of a portion of the Rectum.*—In two cases M. Lisfranc has removed three inches of the lower part of the rectum. The patients have done well.—*Arch. Générales.*

---

*Extraction of Cataract by means of an Incision through the upper part of the Cornea.*—According to Graefe, this method offers numerous advantages over those more usually adopted. The consequences of the wound are less severe, and the sight is more perfectly relieved, because the lower part of the cornea remains untouched, and preserves its natural clearness and convexity. In eighteen persons operated upon by the superior section, seventeen recovered their vision. In one only the cornea on one side became opaque, and this in consequence of a gouty inflammation which frequently returned.—*Bull. des Sciences Med.*

---

### MATERIA MEDICA.

*Variolaria Amera as a Substitute for Quinquinia.*—According to M. Cassebeer, this species of lichen, which grows in abundance on the bark of the beech tree in mountainous forests, possesses a bitter principle similar to that of the quinquina. It results from the experiments tried by the author upon this plant, that it has the same febrifuge properties as the Peruvian bark.—*Magazin für Pharm.*

---

### MISCELLANEOUS.

*Case of Poisoning with Belladonna.*—A man, æt. forty-six, swallowed, by mistake, forty-four grains of the powder of belladonna: an hour afterwards, he was attacked with violent headach, especially over the orbits; the eyes became of a red colour, which quickly extended over the face, and lastly over the body, so that, within a few minutes, the whole skin exhibited an intense uniform redness, such as is observed in scarlet fever; at the same time the patient felt violent pain and heat in the throat, and along the œsophagus; and, on examination, the fauces were found strongly inflamed. These symptoms were accompanied by a very painful irritation of the urinary passages, especially of the neck of the bladder, with a constant but fruitless desire of making water. Copious bleeding, emollient clysters, fomentations on the belly, and twenty-five leeches to the hypogastrium, relieved the patient in some degree, and within twenty-four hours he was perfectly recovered.—*Nouv. Bibl. Méd.*

*Remarks on the Worari and Sirvatan.* By Mr. HANCOCK. (*Quarterly Journal of Science.*)

Centuries have now elapsed since this dreaded weapon, which takes away life like a magic wand, without causing the slightest pang, became known to Europeans, in its effects at least. It is strange, therefore, that the subject should still remain involved in such profound mystery with regard to the poison, the mavacuri plant, which affords it, and that instrument, the sirvatan or blowpipe, through which it is propelled upon the victim.

The question, what plant affords the worari poison, involves, I presume, one of the most interesting inquiries in the whole department of natural history at the present day, and deserves from us a particular and attentive investigation.

Having examined the Mandavacs, Francisco, and Domingo, two intelligent Indians, who were born and bred on the spot, of the tribe most famed for producing the most active worari, and who lived in the vicinity of the mountains which produce both the deadly poison and the instrument of its conveyance, I have received from them separately a most correct and satisfactory account of this affair.

These Indians stated that, both for the mavacuri and sarsa, they go up the Siapo and contiguous streams, or about the mountains of Unturan and of Achivucary, as observed by Humboldt.*

They could give, however, no information respecting the flowers; but they know the plant well, and call it mavacuri; and they state that it is of the gourd kind, or one of the cucurbitacea, of the size of a large orange, round, and having a hard shell or pericarp, which is used at times to contain the poison.

The mahwy, they say, is the plant of which they make the blowpipe for projecting the arrow.

This plant, according to their representation, has large roundish leaves, is jointed, and has slight partitions, like those of the trumpet-tree, which they punch and clear away with long sticks of hard wood, fitted for the purpose. On further conversation with Domingo, it appears to be a species of palm; as, in respect to the texture, leaf, and seed, he compares the different parts to the eta and camawari.

On showing him the small pigmy palm growing on the sands of Essequibo, he said it was the wahwy, exactly in respect to the stem, but not the leaf, as that is bifid; and that it was similarly jointed.

The lining tube is of the same material, a junior or smaller plant of the same kind.

In regard to the manufacture of the poison, Domingo and Fransisco say that they in general add nothing, though some, to thicken it, add the bark. They merely peel or scrape off the bark, and bruise it well in a mortar. The mass is then put into a funnel, or cartocho, made with wild plaintain leaves, and having a little cotton at the bottom to strain it; plenty of cold water is poured over it; and they proceed in the same manner as in drawing the lixivium of ashes. This infusion is put into an earthen pot, (that which is here called a buckpot,) and boiled down to a proper consistence.

This was related circumstantially by Domingo and Francisco, separately.

* They persist that there is no sarsa in Cassiquiari nor in the Rio Negro.

They had no idea of the addition of other substances, (ants, &c.) serving, in reality, only to dilute and render the poison less active, as prescribed by the Indians living near our settlement; all of which are but inventions, like those of the charlatans of Europe, to throw mystery over the affair, and enhance the value of the art. It is very surprising that men of good sense, like Mr. Waterton and Mr. Hillhouse, who, as I should suppose, have had opportunities of better information, should have the credulity to notice or respect such fictions.

The following extract from a letter of Mr. J. Forsyth will throw further light on the subject:

"I received your letter of the 30th ult., requesting a specimen of the worari vine. I am sorry it is not at present in flower; but I send you a small branch of it, and two other vines, called worarybally and courampoey, which the Indians use as auxiliaries to strengthen the former. You will also receive two small roots of the worari vine, which will grow if immediately planted: it will require a great proportion of sand mixed with the earth it is planted in, as it is found growing on sand hills.

"The mode of preparing the poison is as follows: The inner bark or rind of the root (for it is the root only that is used,) is scraped off into some vessel. The worarybally root undergoes the same process; but it is the vine itself of the courampoey that is used. To these, mixed together and well boiled down with some water, the Indians add some peppers, and further boil the whole mass to a thick syrup.

"This account of the process I have had from the Indians; but they are to bring some of these roots, &c. and make the poison in my presence. I shall, therefore, have it in my power, I hope, hereafter to give you a more accurate description of this process."

If such a thing does in reality exist in nature as a direct sedative, in the strictest sense of the term, I should imagine it to be this extraordinary vegetable extract. Its operation on the animal frame is most mysterious. It extinguishes the vital spark without a pang or a struggle, if prepared without any other substance being added; for the most efficient poison is prepared from the worari vine alone. The sensation and effect it produces are extremely analogous to those which arise from excessive bleeding; the animal, under its influence, sinking from existence in the most placid swoon.

On the Parima, amongst the tribes the most celebrated for the use of the worari, I was told that salt and sugar were considered as the best antidotes to this poison. The same was stated to M. de la Condamine, upon the Amazon; but afterwards, if I remember well, it was said to have been disproved by some experiments made in Germany.

I am, nevertheless, inclined to think that some of the tribes do possess a secret antidote to the worari; for I was assured by the Portugueze, that the Indians of the Rio Negro are in the habit of shooting birds and monkeys with the worari, and afterwards resuscitating and transporting them to Para for sale.

This would be an interesting subject for a traveller to investigate. Could such an antidote be found as to render the worari manageable, I feel a persuasion that it would put us in possession of a most important medicinal agent in convulsive disorders, as in tetanus and hydrophobia, and in diseases perhaps of an acute inflammatory nature.

Does it kill by the privation of oxygen, the pabulum of the blood, and supporter of vitality? If this were the *modus operandi* by which it subverts the living powers, its effects might possibly be restrained by inhaling the oxygenous gas, or by cautiously throwing oxygen into the veins.

Be this as it may, it is probable that the same principle belongs to very different plants. If so, an important discovery remains to be made, that of ascertaining the proximate principle which, acting on the nervous and vascular systems, proves so subversive of animal life.

---

*Iodine detected in the Blood.*—M. Bennerscheidt has detected iodine in the crassamentum of the blood of a person who had employed for a long time frictions with iodine ointment. He could not find any indication of its presence in the serum.

---

